# Effects of Early-Life Stress on Social and Anxiety-Like Behaviors in Adult Mice: Sex-Specific Effects

**DOI:** 10.1155/2018/1538931

**Published:** 2018-01-09

**Authors:** Natalya P. Bondar, Arina A. Lepeshko, Vasiliy V. Reshetnikov

**Affiliations:** ^1^Laboratory of Gene Expression Regulation, Institute of Cytology and Genetics, Siberian Branch of the Russian Academy of Sciences, 10 Prospect Lavrentyeva, Novosibirsk 630090, Russia; ^2^Novosibirsk National Research State University, 2 Pirogova Street, Novosibirsk 630090, Russia

## Abstract

Stressful events in an early postnatal period have critical implications for the individual's life and can increase later risk for psychiatric disorders. The aim of this study was to investigate the influence of early-life stress on the social behavior of adult male and female mice. C57Bl/6 mice were exposed to maternal separation (MS, 3 h once a day) or handling (HD, 15 min once a day) on postnatal day 2 through 14. Adult male and female mice were tested for social behavior in the social interaction test and for individual behavior in the plus-maze and open-field tests. Female mice exposed to maternal separation had increased social behavior and increased anxiety. MS male mice had no changes in social behavior but had significantly disrupted individual behavior, including locomotor and exploratory activity. Handling had positive effects on social behavior in males and females and decreased anxiety in males. Our results support the hypothesis that brief separation of pups from their mothers (handling), which can be considered as moderate stress, may result in future positive changes in behavior. Maternal separation has deleterious effects on individual behavior and significant sex-specific effects on social behavior.

## 1. Introduction

There is a wealth of data demonstrating that exposure to stressful events early in life can increase the risk for psychiatric disorders, including mood and anxiety disorders [1–4]. The neural basis of the consequences of early-life stress is poorly understood, and stressful events are supposed to cause structural and functional disturbances in brain regions responsible for emotional behavior in humans [5–7]. Adults with a history of mistreatment in childhood have reductions of medial prefrontal cortex volume [8], decreased hippocampal volume [9, 10], hyperactivity of the hypothalamic-pituitary-adrenal axis [11], and an associated deterioration of sensory and cognitive functions [12]. Animal models of early-life stress also demonstrate disturbances in neuronal activities and brain plasticity [13–17].

Maternal separation (MS) is the most commonly used rodent model of early-life stress [18, 19]. Separation of pups from their dams and nest for 3 or more hours once a day during the first two postnatal weeks produces increased anxiety-like behavior and exaggerated hypothalamic-pituitary-adrenal (HPA)-axis responses to stress in adulthood as well as behavioral and cognitive disturbances [20–24]. These effects have well been studied in rats, while behavioral changes in MS mice remain inconsistent [18, 25].

Nevertheless, consequences of early-life stress in mice are significantly sex-biased. Maternal separation of pups from their dams generally increases anxiety in adult male mice [26–28], while females tend to have a stable anxiety level [25, 27, 29, 30]. Few studies have found increased anxiety in adult female mice after early-life stress [28, 31]. Neither male nor female mice have changes in the level of depression in the forced swim test [25, 26, 32–34]. In mice, MS in combination with restraint stress for mothers during pup separation enhanced the expression of depressive symptoms in the form of decreased sucrose solution preference in both male and female offspring [29]. If maternal separation was combined with early weaning, the authors observed persistent behavioral disturbances in male mice, including enhanced anxiety-like behavior and depressive behavior [35–37]. Apparently, mice require more stress to attain changes in emotion-related behavior and stress reactivity similar to those in rats [25].

In humans, psychiatric disorders such as depression and anxiety are highly comorbid with social dysfunction, and disturbances in social behavior generally appear before these disorders. Therefore, studying dysfunctional social interactions is important for understanding the development of the stress response [38]. However, the influence of maternal separation on social behavior is poorly studied. Studies on males usually find no changes in social behavior in mice [39–41] and rats [42, 43]. A small proportion of the studies reports either slightly intensified social interaction in male mice [29] or, the other way round, decreased social behavior [44] under the influence of early-life stress. Female studies are few, and they provide inconsistent observations: some state that maternal separation results in decreased time investigating other mice [31], while others suggest that it has no effect on social behavior [29]. Thus, effects of early-life stress on adult social behavior remain evasive, especially in their sex-specific aspect.

Interestingly, brief separation of rat pups from their dams (10–15 min per day) often has an opposite effect on later behavioral and stress-related responses compared to prolonged separation [21, 22, 45]. Some mouse studies show decreased anxiety and increased exploratory activity in males [46, 47] and none of these effects in females [25, 30]. The influence of brief separation in early life on mouse social behavior has not yet been investigated.

Here, we attempt to characterize the effects of early-life stress on the social behavior of adult male and female mice and to see if there are correlations between changes and disturbances of individual behavior. A brief (15 min/day) and a prolonged separation (3 h/day) paradigm were used to identify possible positive and negative consequences of early-life adversity.

## 2. Materials and Methods

### 2.1. Animals

C57BL/6J mice were housed in the Center for Genetic Resources of Laboratory Animals (RFMEFI61914X0005 and RFMEFI62114Х0010), Institute of Cytology and Genetics, the Siberian Branch of the Russian Academy of Sciences, Novosibirsk, Russia. The animals were housed under standard conditions (12 : 12 h light/dark cycle, lights on at 8.00 a.m.; feed pellets and water were available ad libitum). All procedures were approved by the Ethics Committee of the Institute of Cytology and Genetics SB RAS (Protocol number 25, December 2014) in conformity with EU Directive 2010/63/EU for animal experiments.

### 2.2. Maternal Separation

**Figure 1:**
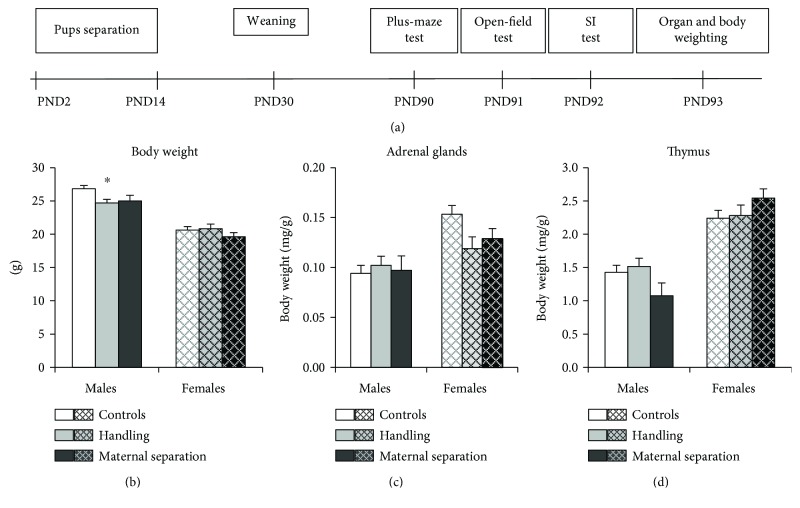
Timeline of the experiment (a), weights of the body (b), relative weights of adrenal glands (c), and thymus (d) in adult male and female mice. Handling decreased the weight of the body in males but not females. Females have the only tendency to reduce relative adrenal weight under early postnatal stress. Relative weights of adrenal glands and thymus are shown as organ weight in mg per gram of body weight. Data present as mean ± standard error of the mean (SEM). White box: controls (males *n* = 11, females *n* = 11); grey box: HD (males *n* = 10, females *n* = 6), black box: MS (males *n* = 6, females *n* = 8); open columns: males; cross-hatched columns: females. ^∗^*p* < 0.05 compared to controls.

Virgin males and females were used for mating. Pregnant females were individually housed with paper nesting material during their third week of gestation. Only litters containing 4–6 pups were used for experiments. All stress procedures were carried out from 1 to 4 pm, in the light phase of day. Offspring were separated from dams on PND 2 through PND 14 (the day of birth was PND 0). At first, each dam was removed from her home cage and placed into a clean cage. Pups were then removed from its home cage and placed together into a small box filled with bedding (one litter for one box). After that, the dam was placed back into the home cage. In the brief separation condition [handling (HD)] the pups were separated from their dams for 15 min once a day, while at the prolonged separation [maternal separation (MS)], the pups were separated for 180 min once a day. The temperature in the MS cages was kept at 31 ± 2°C using infrared heat lamps to prevent thermoregulatory distress. No heat lamps were used on HD pups. The control pups were not separated from their dams. All cages were cleaned on a weekly basis. After weaning on PND 30, the offspring were housed in sibling groups of 2 to 4 animals of the same sex under standard housing conditions. Experimental groups consisted of 27 males (11 control, 10 HD, and 6 MS) and 25 females (11 control, 6 HD, and 8 MS), each group included mice from at least three litters. The behavioral tests were conducted on PND 85–PND 110 in the following order: plus-maze, open-field, and the social interaction test (one test per day). 24 h after the last behavioral test, the animals were euthanized and the adrenal glands and thymus were removed and weighted. Schema of the experiment was presented on Figure 1(a).

### 2.3. Behavioral Tests

#### 2.3.1. Plus-Maze Test

The elevated plus-maze test was performed according to the established procedure [48, 49]. The maze consisted of two opposite open arms (25 cm × 5 cm) and two opposite enclosed arms (25 cm × 5 cm × 15 cm) and had an open roof. The maze was set at 50 cm above the floor. All measurements were made in a dimly lit experimental room. The test apparatus was thoroughly cleaned between tests with different animals. During a 5 min test period, the following parameters of anxiety-like behavior were recorded: the percentage of entries into the open arms, closed arms, and central platform and the percentage of time spent in the open arms, closed arms, and central platform. These parameters were reported as the percentage of total entries and the percentage of testing time, respectively. The additional parameters were the latency of the first exit from the central platform, the number of head-dips, and the number of passages from one closed arm to another.

#### 2.3.2. Open-Field Test

The open field (OF) consisted of a square arena (80 cm × 80 cm) with a white floor and 25 cm high walls. The arena was brightly illuminated and had a central zone (40 cm × 40 cm) and a peripheral zone (anywhere between the central zone and the walls). Each mouse was placed individually in the central zone, and the following behavioral parameters were recorded during a 5 min test period: the total distance traveled; the latency of the first exit from the central zone; the number of visits to the central zone; the time spent in the central and the peripheral zone; the number of rearing and self-grooming episodes; and total activity state (activity level of mouse was determined by the software as a function of pixel changes, EthoVision XT threshold for activity—0.2%). The OF arena was thoroughly cleaned between tests with different animals.

#### 2.3.3. Social Interaction Test

A square plastic arena (40 cm × 40 cm × 25 cm) was used for the social interaction test. A small perforated plastic target box (10 cm × 10 cm) was placed near the center of one of the walls. The test consisted of two trials (5 min each). At the beginning of each trial, an experimental mouse was placed in the zone opposite to the target box, face to the wall. In the adaptation trial, the target box was empty and the animal was habituated to the novel environment for 5 min. In the interaction trial, an unfamiliar partner of the same sex was placed into the target box and the reaction of the experimental mouse to the social target was investigated. Between the trials, the mouse was placed to a neutral cage. The following parameters were recorded during each trial: the total distance traveled, the number of visits to the interaction zone (a 5 cm zone around the target box), the time spent in the interaction zone, and the latency of the first contact with the target box. The interaction ratio was calculated as 100^∗^(time in the interaction zone, partner present)/(time in the interaction zone, partner absent).

### 2.4. Characterization of Estrous Phase

Vaginal smears from each female were taken every day after behavioral tests to determine the phase of the estrous cycle. Based on vaginal cytology, females were divided into two groups: diestrous (those in diestrus and metaestrus) and estrous (those in estrus and proestrus).

### 2.5. Statistical Analysis

All tests were videotaped and manually scored by the free open-source software BORIS (Behavioral Observation Research Interactive Software, http://www.boris.unito.it) [50]. The distance traveled in the OF test was scored using EthoVision XT v.10.0 (Noldus Information Technology, Netherlands).

Normal distribution and homogeneity of variances were tested using Shapiro–Wilk's and Levene's tests, respectively. As most behavioral data were not normally distributed, nonparametric tests were used. The statistical analysis of behavioral data was performed using Kruskal–Wallis one-way ANOVA, with the type of stress as a factor separately for males and females. Pairwise comparisons were performed by the Mann–Whitney *U* test. The statistical significance threshold was set at *p* < 0.05.

## 3. Results

### 3.1. Body, Adrenal Glands, and Thymus Weight

Males had more body weight and less relative weights of adrenals and thymus than females (effect of sex: for body weight *H* (1, 52) = 18.8, *p* < 0.001; for adrenals *H* (1, 52) = 29.1, *p* < 0.001; for thymus *H* (1, 52) = 35.9, *p* < 0.001) (Figure 1). Early postnatal stress has an effect on body weight in males (*H* (2, 27) = 6.8, *p* = 0.03); in HD males, the body weight is reduced in comparison with the control (*p* = 0.017), and MS males also have a tendency to reduce the body weight (*p* = 0.0841). Females have the only tendency to reduce relative adrenal weight under early postnatal stress (*H* (1, 19) = 3.3, *p* = 0.069).

### 3.2. Plus-Maze Behavior

Generally, males were less anxious than females (more time spent in open arms (*H* (1, 52) = 6.6, *p* = 0.01) and center (*H* (1, 52) = 19.9, *p* < 0.001)) and have higher locomotor activity than females (more total number of entries (*H* (1, *N* = 52) = 15.9, *p* = 0.0001); more passages (*H* (1, *N* = 52) = 6.2, *p* = 0.0126)).

**Figure 2:**
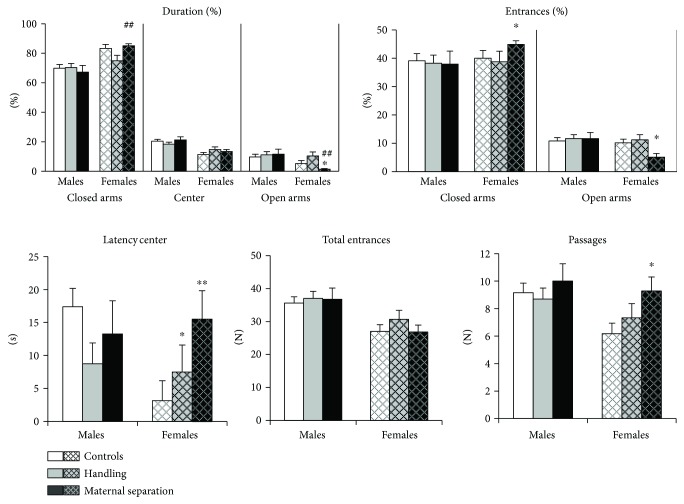
Effects of early-life stress on the behavior of adult mice in the plus-maze test. Early-life stress had no effect on plus-maze behavior in male mice but led to significant anxiogenic effect in MS females. Data present as mean ± standard error of the mean (SEM). White box: controls (males *n* = 11, females *n* = 11); grey box: HD (males *n* = 10, females *n* = 6), black box: MS (males *n* = 6, females *n* = 8); open columns: males; cross-hatched columns: females. ^∗^*p* < 0.05, ^∗∗^*p* < 0.01 compared to controls; ^##^*p* < 0.01 compared to HD.

Early-life stress had no effect on plus-maze behavior in male mice (Figure 2). However, stress in early life led to significant anxiogenic effect in MS females. MS females had increased latency to exit the central platform compared with controls (effect of stress: *H* (2, 24) = 9.9, *p* = 0.007; Mann–Whitney *U* test: *U* = 8, *p* = 0.006) and spent less time in the open arms than controls (effect of stress: *H* (2, 24) = 8.6, *p* = 0.013; Mann–Whitney *U* test: *U* = 16, *p* = 0.041) and HD mice (*U* = 3, *p* = 0.01). Stress has a weak effect on the number of entries into the closed (a trend, *H* (2, 24) = 5.1, *p* = 0.077) and open (a trend, *H* (2, 24) = 5.1, *p* = 0.077) arms. Nevertheless, the percentage of entries into the open arms was decreased (*U* = 17, *p* = 0.05) and the percentage of entries into the closed arms was increased (*U* = 17, *p* = 0.05) in MS females compared to controls. Anxious effect was not caused by a decrease in locomotor activity of females (total entries, *p* > 0.05), and MS females even had a higher number of passages between the closed arms compared to controls (*U* = 12.5, *p* = 0.018). HD females had only increased latency to exit the central platform compared with controls (*U* = 11.5, *p* = 0.031).

**Figure 3:**
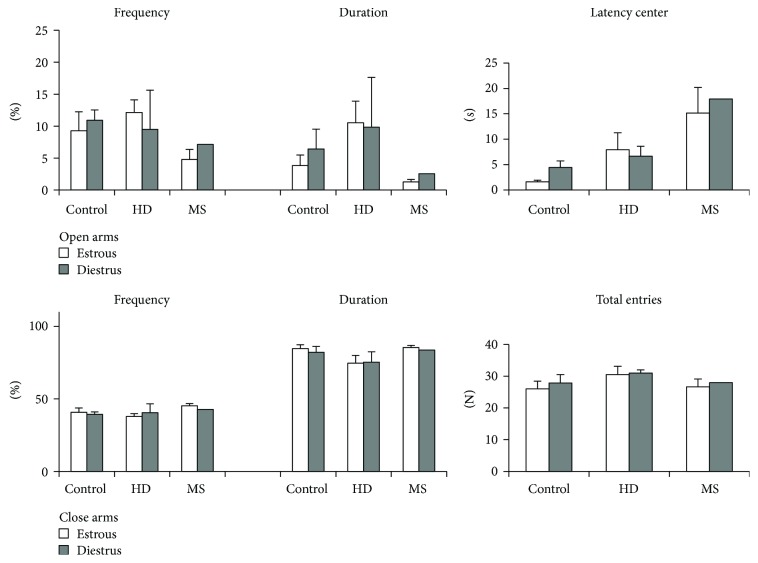
The influence of early-life stress on the behavior of adult female mice in plus-maze test in different phases of estrous cycle. Data present as mean ± standard error of the mean (SEM). White box: estrous (5 female controls, 4 HD group, and 6 MS group), gray box: diestrus (6 female controls, 2 HD group, and 1 MS group). Insufficient number of mice in each phase did not allow a correct statistical analysis.

Females were grouped by the phase of the estrous cycle to assess its effect on plus-maze behavior. However, an insufficient number of mice in each phase does not allow a correct statistical analysis. MS females tended to have increased anxiety both in estrus and diestrus (Figure 3).

### 3.3. Open-Field Behavior

Males have higher locomotor activity than females (longer distance travelled: *H* (1, 52) = 17.8, *p* < 0.001, higher total activity: *H* (1, 52) = 24.7, *p* < 0.001) and more often go to the central zone (*H* (1, 52) = 9.3, *p* = 0.002), indicating that males are less anxious than females in OF.

#### 3.3.1. Males

**Figure 4:**
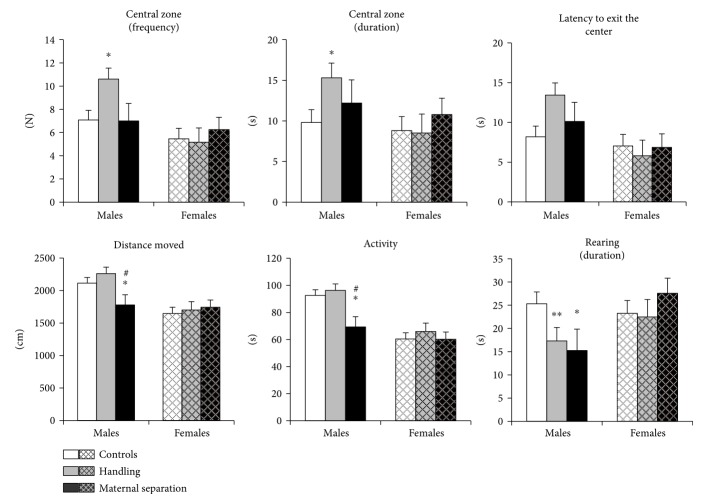
Effects of early-life stress on the behavior of adult mice in the open-field test. Male mice showed decreased locomotor and exploratory activities of MS males and decreased anxiety-like behavior in HD males. Females did not show the behavioral changes in the test. Data present as mean ± standard error of the mean (SEM). White box: controls (males *n* = 11, females *n* = 11); grey box: HD (males *n* = 10, females *n* = 6), black box: MS (males *n* = 6, females *n* = 8); open columns: males; cross-hatched columns: females. ^∗^*p* < 0.05, ^∗∗^*p* < 0.01 compared to controls; ^#^*p* < 0.05 compared to HD.

In the OF test, maternal separation had the greatest effect on the locomotor and exploratory activities of animals (Figure 4). Stress had an influence on total activity (*H* (2, 27) = 6.5; *p* = 0.038); parameters reflecting any movements of mice and the total activity of MS males were lower than in the control (*U* = 7.0; *p* = 0.031) and HD group (*U* = 3.0; *p* = 0.016). Decrease in activity is accompanied by decreasing distance travelled (stress effect: *H* (2, 27) = 5.0; *p* = 0.083; *U* = 9.0; *p* = 0.05 compared to control; *U* = 6.0; *p* = 0.048 compared to the HD group). The MS group has less the duration of rearing (*H* (2, 27) = 8.6; *p* = 0.013; *U* = 8.0; *p* = 0.04) than the controls that reflects a decrease exploratory activity under prolonged postnatal stress.

Brief postnatal stress (handling) has an effect, mainly, on anxiety-like behavior. The number of visits (effect of stress: *H* (2, 27) = 5.6; *p* = 0.062; *U* = 28.5; *p* = 0.022) and time spent in the central zone (effect of stress: *H* (2, 27) = 5.2; *p* = 0.075; *U* = 25.0; *p* = 0.013) in the HD group were increased to compare with controls, reflecting a slight decrease in anxiety. Moreover, the duration of rearing were decreased in the HD groups compared to controls (*U* = 23.0; *p* = 0.009), as in the MS group. Parameters of locomotor activity were unchanged in the HD group.

#### 3.3.2. Females

**Figure 5:**
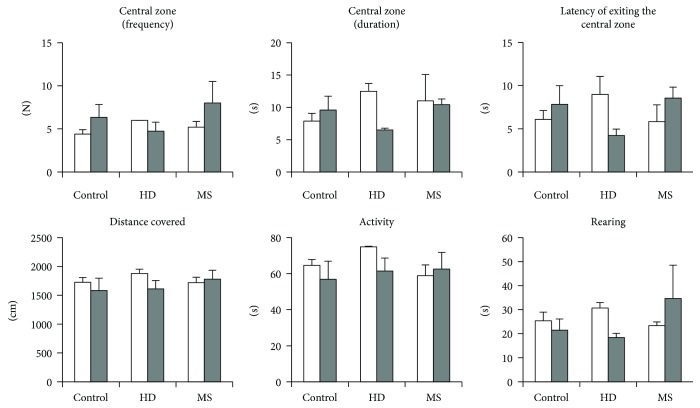
The influence of early-life stress on the behavior of adult female mice in open-field test in different phases of estrous cycle. Data present as mean ± standard error of the mean (SEM). White box: estrus (5 female controls, 2 HD group, and 5 MS group), gray box: diestrus (6 female controls, 4 HD group, and 3 MS group). Insufficient number of mice in each phase did not allow a correct statistical analysis.

The analysis did not reveal any significant differences between groups (Figure 4). Estrous phase did not influence behavior in the OF test. However, diestrous HD females were slightly more anxious than MS females (Figure 5).

### 3.4. Social Interaction Test

Social interaction test assesses the level of sociability, which is measured by comparing the time a mouse spends in an interaction zone with a social target to the time in that zone in the absence of a social target. Males spend more time than females in the interaction zone both with an empty box and with a social target (effect of sex: *H* (1, 52) = 6.2, *p* = 0.012; *H* (1, 52) = 4.6, *p* = 0.033, resp.).

#### 3.4.1. Males

**Figure 6:**
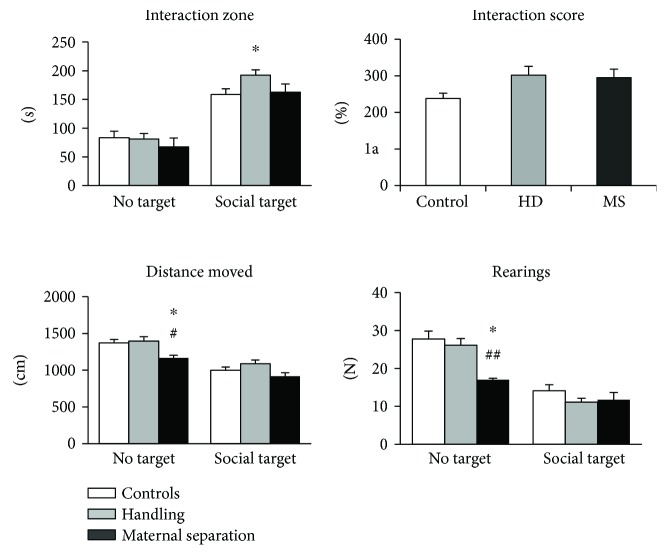
Effects of early-life stress on the social behavior of adult male mice. Handling have increased sociability of males, whereas maternal separation had no effects on social behavior. Data present as mean ± standard error of the mean (SEM). White box: controls (males, *n* = 11); grey box: HD (males, *n* = 10), black box: MS (males, *n* = 6); no target: the adaptation trial with an empty target box; social target: the interaction trial with an unfamiliar partner in the target box. ^∗^*p* < 0.05 compared to controls; ^#^*p* < 0.05; ^##^*p* < 0.01 compared to HD.

Behaviors in an adaptation trial of the SI test, when a mouse can investigate a new place, confirm the decline locomotor and exploratory activities in the MS group. MS males covered significantly less distance than controls (effect of stress: *H* (2, 27) = 5.5, *p* = 0.064; *U* = 8.0; *p* = 0.042) and HD males (*U* = 4.0; *p* = 0.024) (Figure 6). The number of rearing was lower in MS males than in controls (effect of stress: *H* (2, 27) = 8.1, *p* = 0.017; *U* = 4.0; *p* = 0.012) and HD males (*U* = 0.5; *p* = 0.006).

In the interaction trial, when an unknown mouse was introduced into the small box, all groups had increased time spent in the interaction zone (interaction score > 100%, Figure 6). A significant effect of stress was revealed on the time spent in the interaction zone (*H* (2, 27) = 8.1, *p* = 0.018); HD males spent more time responding to the social target than controls (*U* = 30.0; *p* = 0.03). The MS group did not differ from controls and the HD group.

#### 3.4.2. Females

**Figure 7:**
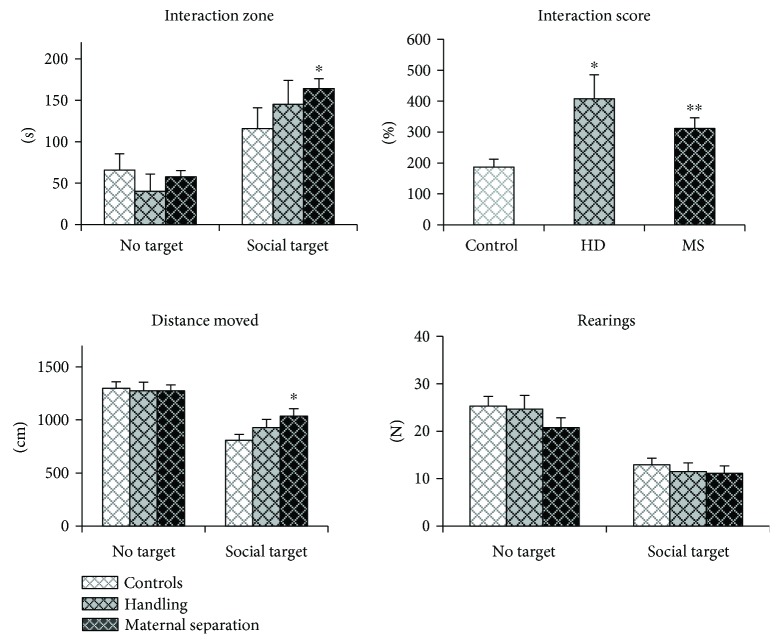
Effects of early-life stress on the social behavior of adult female mice. Both stressed females groups increased sociability in the test. Data present as mean ± standard error of the mean (SEM). White box: controls (females *n* = 11); grey box: HD (females *n* = 6), black box: MS (females *n* = 8); no target: the adaptation trial with an empty target box; social target: the interaction trial with an unfamiliar partner in the target box. ^∗^*p* < 0.05, ^∗∗^*p* < 0.01 compared to controls.

Early-life stress has no significant effect on behavioral parameters in the adaptation trial. In the interaction trial, all groups had increased time spent in the interaction zone when a partner was presented (interaction score > 100%, Figure 7). Surprisingly, the interaction scores in HD and MS females were higher than in controls (effect of stress: *H* (2, 25) = 10.3, *p* = 0.006; *U* = 8.0; *p* = 0.012 and *U* = 11.0; *p* = 0.006, resp.), indicating that both stressed females groups increased sociability. MS females have enhanced not only score but also time spent near the social target compared to controls (effect of stress: *H* (2, 25) = 4.7, *p* = 0.096; *U* = 17.0; *p* = 0.026). And, apparently, this increased sociability led to an increase in the distance traveled in MS females compared to controls (effect of stress: *H* (2, 25) =6.0, *p* = 0.049; *U* = 17.0; *p* = 0.026).

**Figure 8:**
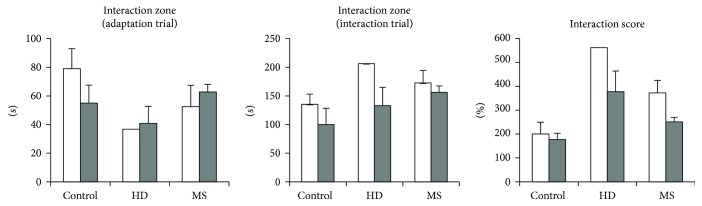
The influence of early-life stress on the behavior of adult female mice in social interaction test in different phases of estrous cycle. Data present as mean ± standard error of the mean (SEM). White box: estrus (5 control females, 1 HD group, and 4 MS group), gray box: diestrus (6 control females, 5 HD group, and 4 MS group). Insufficient number of mice in each phase did not allow a correct statistical analysis.

Figure 8 shows behavioral parameters depend on estrus and diestrus. There was only one estrous HD female in the test, which complicated the statistical analysis of the estrous cycle.

## 4. Discussion

Our study focused on the effects of maternal separation on the social behavior of adult mice and compared changes with changes in individual behavior. We showed that early postnatal stress had different effects on males and females.

The study of individual female behavior in the MS group revealed significantly increased anxiety in the elevated plus maze. Females avoided the open arms of the maze, but their locomotor activity was the same as that of controls. The OF test also did not reveal changes in the locomotor activity of MS females. In contrast to females, MS male anxiety in our experiments remained unchanged. The lack of MS effect on male emotional behavior was accompanied by changes in locomotor and exploratory activity. The OF test showed a significantly less distance traveled and reduced total and exploratory activity. Moreover, less distance was covered in the SI test during the adaptation trial, when the mice explore a new field without any additional social stimulus. So we have found gender-specific influence of prolonged maternal separation on anxiety and locomotion. There are few works in which both males and females of the С57BL6 mice were simultaneously studied. Some of them found the same effects of MS on anxiety in males and females [28, 30], whereas one study revealed sex-specific influence on anxiety [27], but different from our results. Various results can be explained by the differences in methodology of maternal separation. So, for example, in the study of Romeo and colleagues [27], separated pups were kept at the ambient temperature of the vivarium during 3 hours without additional heating that can lead to a pup's hypothermia. Some of the works used sexually experienced females (having two or three litters before) [25, 30]. Procedures for checking health and gender of pups as well as culling the pups at first postnatal day are stress factors for dams and pups and can affect results of the experiment [28]. So, minor differences in methodological aspects lead to a significant disagreement in the results.

These disagreements and other inconsistent results [31, 32, 51–55] suggest that effects on anxiety in mice are highly variable and depend even on small variations in environmental conditions during the postnatal period. Similarly, Millstein and Holmes [25] have concluded that repeated postnatal MS did not produce clear changes in anxiety- or depression-related behaviors in any of the five mouse strains studied. However, even in the lack of MS effects on behavior, it may alter the feedback regulation of response to a subsequent stress event, for example, resulting in a more pronounced and prolonged elevation of corticosterone levels [56–58] or a greater decrease in GR expression in the frontal cortex and hippocampus [59, 60].

The study of social behavior revealed somewhat unexpected results in females with prolonged maternal separation. The social interaction test showed increased sociability in MS females: the females responded to a novel conspecific more actively than the controls. However, this is not consistent with a high level of anxiety observed in females given the plus-maze test. High anxiety normally inhibits exploratory or social activity in animals. Here, we can hypothesize that the adaptation trial, during which the animals are familiarizing themselves with the experimental field, reduces anxiety. As is known, animals in familiar situations are less anxious and tend to have longer social contacts [61]. Additionally, longer testing decreases the fear of new space and stimulates exploratory behavior [62, 63]. As is known, anxiety in new, unfamiliar situations (“state” anxiety) does not necessarily correlate with anxiety shown in familiar situations (“trait” anxiety) [64, 65]. Some kinds of stress, for example, repeated aggression experience, induce enhanced anxiety [66] as observed in the plus-maze together with enhanced exploratory activity towards a new neutral stimulus [67]. It is possible that early-life stress in MS females increases “state,” but not “trait” anxiety, and that these females, when in a familiar situation, can display a high level of social behavior. However, this hypothesis requires scrutiny.

Interestingly, in contrast to females, the social behavior of adult males was absolutely unaffected by prolonged maternal separation, and the level of reaction to the presentation of another mouse remained the same as in controls, which is consistent with some other social behavioral studies in males [39, 40].

Thus, in our experiment, separating the pups from their dams for 3 hours in an early postnatal period has a pronounced sex-specific effect on both social and individual mice behavior. Females are more sensitive to stress than males, and maternal separation has contrasting effects (increased anxiety versus increased sociability) on the former.

Results of rat experiments demonstrated that brief maternal separation, or handling, which can be considered moderately stressful for pups, can result in positive behavioral changes and even tolerance to more intensive stress [21, 22, 45, 56, 68]. In our experiment, we also have found a positive effect of handling—HD males demonstrated a decreased anxiety in the OF test and increased sociability in the SI test. Males were not afraid of visiting the central part of the open field and spent more time there than the controls or the MS group, and HD males also spent more time in the interaction zone when the unknown partner was presented. Brief separation of females had no effect on anxiety or individual and exploratory behavior but also an increased level of sociability—HD females had the highest interaction score, which reflects the difference between a reaction to a partner and a reaction to an empty box. There are few works on the impact of handling on subsequent behavior of adult mice. Unlike rat studies, the vast majority of studies of HD stress have not found significant changes in anxiety or individual behavior in male and female C57BL6 mice [25, 30, 69]. Only one study has found decreased anxiety in male mice induced by brief separation [46], which is similar to our results. Our study has shown for the first time, to our knowledge, that brief maternal separation may positively affect social behavior in mice. Both males and females showed increased sociability in the form of a more active reaction to the same-sex partner in the social interaction test.

Our study has shown that female mice have more pronounced positive and negative consequences of both maternal separation and neonatal handling compared to their male conspecifics. Compared to male rodents, female rodents are known to have greater corticosterone release under basal conditions [70] and in response to stress [71–74]. Female mice were shown to be more sensitive to unpredictable chronic mild stress [75], subchronic variable stress [76], and even acute stress [77]. Also, females have longer consequences of social defeat stress than males [78, 79]. These findings explain the higher sensitivity of females to early-life stress consequences.

According to a systematic review [18], female studies nearly always show positive or deleterious effects of early stress on anxiety, depressiveness, or cognitive functions, while male studies do not reveal significant effects of stress on these psychoemotional traits in about 30% of cases. The analysis of maternal separation consequences [18] suggests that, in most (but not all) cases, stress results in higher anxiety and cognitive deterioration in both males and females. However, a sex-specific difference is sometimes observed in the studies. The study by Romeo and colleagues [27] showed opposite changes in the locomotor activity of males and females, and another study [28] identified contrasting changes in aggressive behavior. Increased social behavior after maternal separation that we have discovered is typical of females only, while males have the same reaction to the partner as controls. Notably, brief separation has the same effect on social behavior both in males and females. Thus, the influence of early-life stress on behavior in adulthood is gender-dependent.

Multiple studies in sex hormone effects on emotional behavior and stress response [80, 81] suggest that estrous cycle should be taken into account in female experiments. Although emotional behavior and especially the level of anxiety in females are sensitive to the level of sex hormones [82, 83], only few studies on female behavior after early-life stress [27, 84] take into account the phase of the estrous cycle. Most researchers believe that daily vaginal smears are too stressful for animals or that the selections of females used in the studies are insufficiently representative to analyze [25, 28–30]. However, studies often find no correlation between behavior and cycle phase [85, 86]. The reason may be that hormonal influence is not very strong, and the stress of testing hides possible behavior dependence on hormones. We have not detected an influence of cycle phase on the behavioral traits studied. Proper statistical analysis was impeded by a low number of animals in the groups. However, the direction of change was the same in the estrous and diestrous phases, that is, hormonal level had an insignificant effect on emotional behavior, locomotor, and exploratory activity.

## 5. Conclusion

Thus, the present study shows that the social and individual behavior of adult mice can be modified by early-life events in a sex-specific manner. We have found no changes in the anxiety or social behavior of MS males. Disrupted individual behavior has been the only consequence of early stress. The intensity of stress may have been insufficient to produce later effects in males. Females have been more sensitive to stress. Maternal separation has led to increased female anxiety and social behavior. Brief separation of pups from their mothers resulted in positive behavioral changes in males and females. Both sexes had increased social behavior, and males had decreased anxiety, which confirms the hypothesis that mild early-life stress has a positive effect on later emotional behavior.
